# Serum troponin, D‐dimer, and CRP level in severe coronavirus (COVID‐19) patients

**DOI:** 10.1002/iid3.582

**Published:** 2021-12-22

**Authors:** Ayad M. Ali, Hassan M. Rostam, Mohammed H. Fatah, Chalak M. Noori, Kameran M. Ali, Hassan M. Tawfeeq

**Affiliations:** ^1^ Department of Chemistry College of Sciences, University of Garmian Kalar Kurdistan Region Iraq; ^2^ Immunology & Immuno‐Bioengineering Group, School of Life Sciences, Faculty of Medicine & Health Sciences, University of Nottingham Nottingham UK; ^3^ Department of Medicine, College of Medicine University of Garmian Kalar Kurdistan Region Iraq; ^4^ Medical Lab Technology Department, Kalar Technical College Sulaimani Polytechnic University Kalar Kurdistan Region Iraq; ^5^ Department of Law College of Law and International Relation, Lebanese French University Erbil Kurdistan Region Iraq

**Keywords:** COVID‐19, CRP, D‐dimer, SARS‐CoV‐2, troponin

## Abstract

**Background:**

Abnormal inflammation coagulation biomarker levels of troponin, C‐reactive protein (CRP), and D‐dimer levels in serum have been demonstrated to be associated and involved in the disease progression of coronavirus disease 2019 (COVID‐19).

**Methods:**

First: the study aimed to investigate the correlation of troponin, CRP, d‐dimer, white blood cell (WBC) and polymerase chain reaction–cycle threshold (PCR‐Ct) within COVID‐19 survivors (143 patients; 79 males, 64 females) and in deceased (30 patients; 12 males, 18 females) group. Also, assessing any differences between both groups in studied parameters. Second: a correlation study of studied parameters' level has been conducted within families (41 patients; 23 males [seven deaths] and 18 females [eight deaths]) that lost more than one member due to the severity of the disease. Also, differences between these family and control group (132 patients; 69 males and 63 females) group in studied parameters have been assessed.

**Results:**

In the first week of hospitalization, there were significant differences in D‐dimer, CRP and troponin level between survived and deceased patient groups. In the second week of the admission, both groups had significant differences in the level of all studied parameters; troponin I, D‐dimer, CRP, and WBCs. WBC levels positively correlated to CRP in male survivors (*r* = 0.75, *p* < 0.0001), and to troponin in deceased male patients (*r* = 0.74, *p* = 0.007). The second week of patient admission was critical in the group of families who lost more than one person, when troponin was correlated positively with D‐dimer, CRP, and WBCs.

**Conclusion:**

Troponin, D‐dimer, CRP, and WBCs level were significantly higher in COVID‐19 patients who died than in COVID‐19 survivors. High troponin and WBC levels, were considerably associated with families that lost more than one member, when compared with the unrelated COVID‐19 patient control.

## Graphical abstract

Troponin, D‐dimer, C‐reactive protein (CRP), and white blood cells (WBCs) level were significantly higher in COVID‐19 patients who died than in COVID‐19 survivors. High troponin and WBC levels, were considerably associated with families that lost more than one member, when compared with the unrelated COVID‐19 patient control.

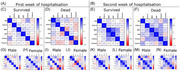

## 1 INTRODUCTION

Beginning in December 2019, the highly infectious coronavirus disease 2019 (COVID‐19), mainly involving respiratory failure associated with severe acute respiratory syndrome coronavirus 2 (SARS‐CoV‐2), has emerged as a most threatening worldwide pandemic.^1^, ^2^ As of June 10th, 2021, the World Health Organization announced approximately 175 million confirmed COVID‐19 cases and 3.8 million deaths worldwide.^3^ COVID‐19 patients mostly show flu‐like symptoms such as fever, cough, myalgia, dyspnoea, and fatigue.^4^ According to their clinical manifestation, COVID‐19 patients are classified as mild, moderate, severe, or critical.^5^ Serious cases of the disease can lead to severe pneumonia, respiratory dysfunction, multiple organ failure, and, subsequently, death.^6^

There are several biomarkers associated with the severity of COVID‐19, including C‐reactive protein (CRP), D‐dimer, and troponin.^7^ COVID‐19 patients who die exhibit higher levels of troponin I, white blood cells (WBCs), d‐dimer, and CRP when compared with COVID‐19 survivors.^8^ Thus, prognostic levels of biomarkers associated with COVID‐19 may assist in understanding the progression of the disease.^9^ A significant increase in troponin due to cardiac injury in COVID‐19 has been correlated with risk of mortality,^10^ which may be due to ACE2 expression by endothelial and myocardial cells.^11^ Although the biological mechanisms underpinning the elevated levels of plasma D‐dimer associated with COVID‐19 remain poorly understood, initial studies from Wuhan suggest that the increase in D‐dimers is associated with disseminated intravascular coagulopathy.^12^ Furthermore, it is now well known that severity and mortality in COVID‐19 are positively correlated with age.^11^ As evidence, a higher COVID‐19‐related mortality rate has been observed in individuals over the age of 65 years when compared to those who are younger, particularly those under 25 years of age, regardless of gender differences.^13^ Additionally, the correlation between COVID‐19 fatality and family genetics is not fully understood, although one study reported higher mortality from COVID‐19 in families possessing the human leukocyte antigen (HLA) gene alleles HLA‐A*11, C*01, and DQB1*04.^14^

Here, we performed a retrospective study to evaluate any correlation of the potential risk factors troponin, D‐dimer, CRP, WBCs, and reverse‐transcription polymerase chain reaction–cycle threshold (PCR‐Ct) in COVID‐19 patients in families with higher fatality and in other COVID‐19 patients. We also compared the levels of those parameters between families with higher mortality and other COVID‐19 patients. Additionally, correlations among some parameters, such as age, PCR‐Ct, WBCs, CRP, D‐dimer, and troponin, in COVID‐19 patients in general were also investigated. Furthermore, correlations among the studied parameters were examined for survivors and deceased patients, taking into account the effect of gender in each group. Finally, the differences in those parameters between survivors and non‐survivors were studied and compared between the first and second weeks of admission.

## 2 MATERIALS AND METHODS

### 2.1 Study design and patients

**Figure 1:**
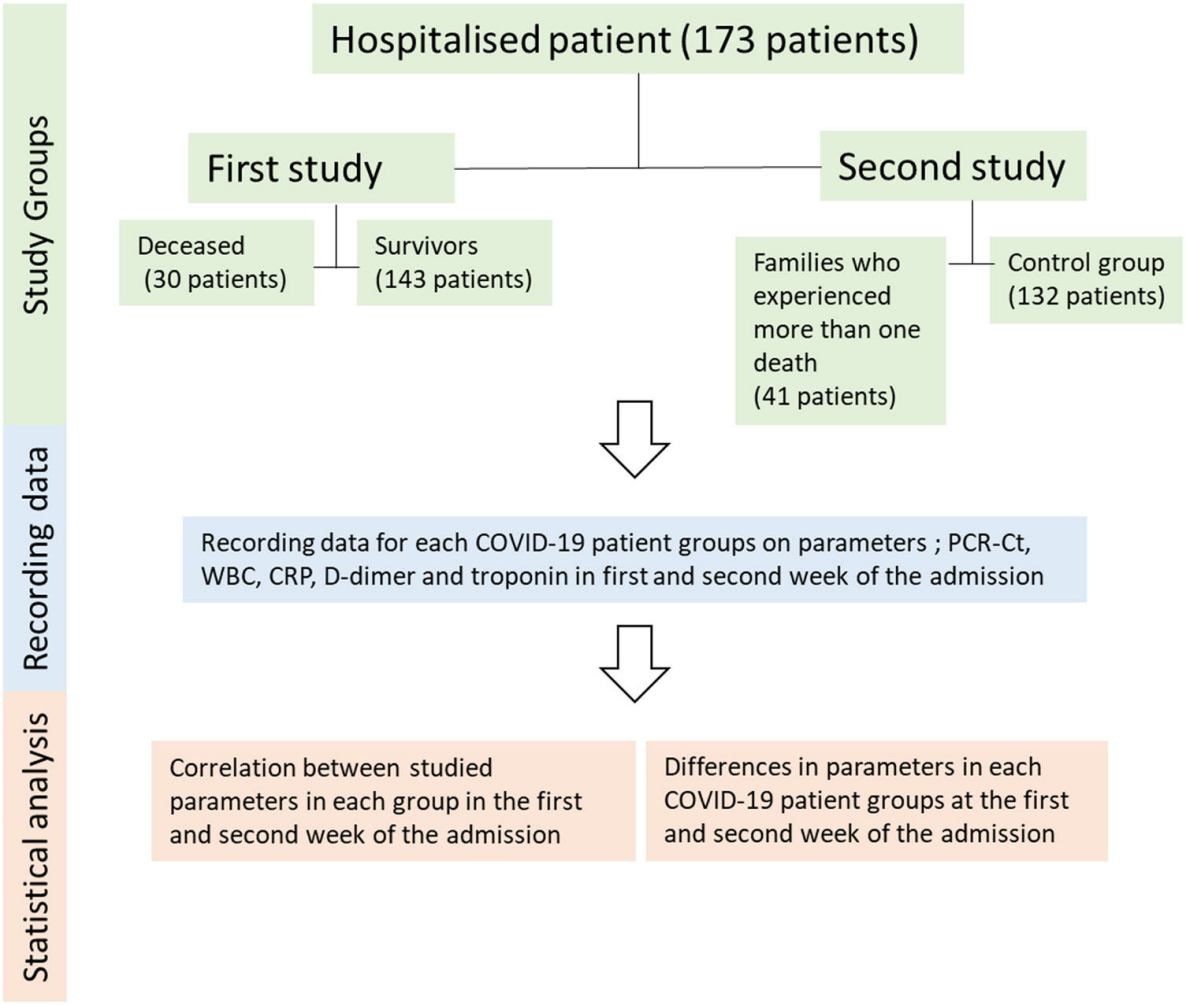
Flowchart about the study design and steps

The first study included 173 hospitalized (severe) COVID‐19 confirmed patients (91 males, 82 females) who were divided into two categories: 143 survivors (79 males, 64 females) and 30 deceased (12 males, 18 females). The same patients were re‐categorized in the second study into two different categories: seven families who experienced more than one death in hospital (41 patients), and a control group of 132 other hospitalized confirmed cases (Figure 1). The study was conducted at Qala Hospital for COVID‐19, Kalar, Kurdistan Region, Iraq from August 21, 2020 to March 6, 2021.

According to the *Novel Coronavirus Pneumonia Diagnosis and Treatment Protocol* (Seventh Edition) of the National Health Commission,^15^ the clinical classification at admission consisted of one of three types: mild cases—mild clinical symptoms with no pneumonia on imaging; moderate cases—fever with respiratory symptoms and presence of pneumonia on imaging; and severe cases, which included adults with any of the following—respiratory distress, respiratory rate ≥30 breaths/min, oxygen saturation of ≤93% during inhalation at rest, and/or a ratio of arterial partial pressure of oxygen to fraction of inspired oxygen of ≤300 mmHg.

### 2.2 Reverse‐transcription polymerase chain reaction (RT‐PCR)

Real‐time RT‐PCR to detect SARS‐CoV‐2 was performed using a viral RNA template extracted from nasopharyngeal swab samples. After collection, the total RNA was automatically isolated within 45 min using the Qiagen EZ1 Advanced XL system (Qiagen). The presence of SARS‐CoV‐2 was then detected by RT‐PCR amplification of fragments of the SARS‐CoV‐2 open reading frame 1ab (ORF1ab) and envelope (E) genes, using the PowerChek SARS‐CoV‐2 Real‐Time PCR Kit (KogeneBiotech). Thermocycling was performed on a Rotor‐Gene Q thermocycler (Qiagen), with the conditions for amplification being 50°C for 30 min, 95°C for 10 min, 40 cycles of 95°C for 15 s, and then 60°C for 1 min. When the two target genes (ORF1ab and E) tested positive by specific real‐time RT‐PCR, a cycle threshold value (Ct‐value) ≤ 36.7 was defined as a positive result and a Ct‐value of greater than 36.7 was defined as a negative result.^16^ ^,^ ^17^Ct‐value is a semi‐quantitative assessment that can widely show the concentration of viral genetic material in the patient samples following testing by RT‐PCR. A low Ct‐value indicates a high concentration of viral genetic material, which is typically associated with a high risk of severity, but a high Ct‐value indicates a low concentration of viral genetic material which is typically associated with a lower risk of the disease severity.^18^

### 2.3 Biological markers

The biological marker tests conducted in this study included complete blood count (CBC), CRP, D‐dimer, and troponin I. The CBC was primarily performed using a Medonic M‐Series haematology analyzer (Medonic M32; Boule Medical AB) and quantitative immunological determination of CRP in serum was performed using the cobas c111 (Roche Diagnostics) system. Measurement of CRP aids in evaluating the amount of inflammation, with normal values for adults being <5.0 mg/L. Fluorescence immunoassay ichroma™ II (Boditech Med Inc.) was used for the D‐dimer Rapid Quantitative Test, with a normal value being <500 ng/ml. Cardiac troponin I tests were analyzed by the cobas e 411 analyzer (Roche Diagnostics), which is a fully automated analyzer that uses patented ElectroChemiLuminescence technology for immunoassay analysis. The normal range for troponin is between 0 and 0.4 ng/ml.

### 2.4 Statistical analysis

Pearson correlation and polynomial regression were employed to analyze the correlations among troponin I, d‐dimer, CRP, WBCs, and PCR‐Ct in each studied COVID‐19 group. One‐way ANOVA was used to analyze differences in the investigated parameters between the two groups.

## 3 RESULTS AND DISCUSSION

### 3.1 First study

**Figure 2:**
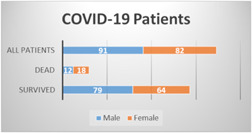
First studied COVID‐19 patients, including hospitalized 173 patients (male, 91 and female, 82). Studied COVID‐19 patients divided into two categories; survivors patients, 143 (male, 79 and female, 64) and dead patient, 30 (male, 12 and female, 18)

The first part of the current study was conducted on 173 COVID‐19 hospitalized (severe) patients, of which 91 (52.6%) were male and 82 (47.4%) were female. This gender infection rate with SARS‐CoV‐2 is similar to that of other studies.^19^, ^20^, ^21^ The first study group was divided into two categories: survivors (143; 79 males, 64 females) and deceased (30; 12 males, 18 females; Figure 2).

The rate of death among females (18/82; 21.9%) in the current study was generally higher than that of males (12/91; 13.1%). This result conflicts with data from a study conducted with more than 1500 COVID‐19 patients from different areas of the world, which concluded that male patients have a higher mortality rate than females.^19^ This discrepancy may be due to age, as the females in the current study were older (60.16 ± 12.11 years) than the males (59.35 ± 10.25 years), and older patients are more vulnerable to severe disease and death from SARS‐CoV‐2 infection.^22^, ^23^

**Figure 3:**
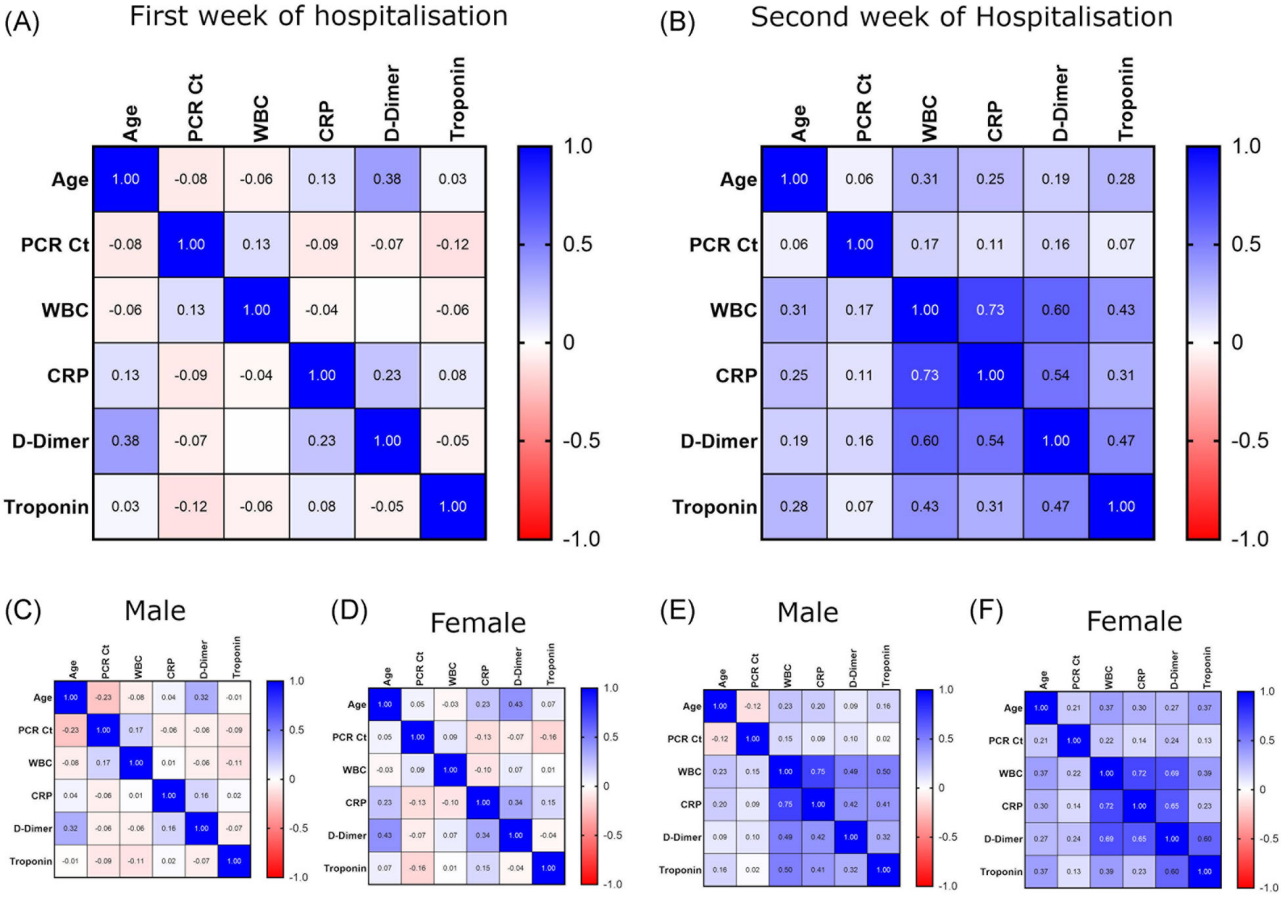
Correlation between studied parameters; Age, PCR‐Ct, WBC, CRP, d‐dimer and troponin at the COVID‐19 patients first week of admission (A) in male (C) and female (D). Also at the second week of admission (B) in male (E) and female (F). CRP, C‐reactive protein; PCR‐Ct, polymerase chain reaction–cycle threshold; WBC, white blood cell

In the first week of admission to the hospital, there was no correlation among any studied parameters (Figure 3A). This was likely because the immune system requires sufficient time to react specifically to a disease, so when the body encounters the SARS‐CoV‐2 virus for the first time, the immune system cannot work properly and illness may then occur.^24^

In the second week of hospitalization, we found a positive correlation (*r* = 0.73, *p* < 0.0001) between WBCs and CRP (Figure 3B). When patients were divided by gender, the positive correlation remained (male, *r* = 0.75, *p* < 0.0001; female, *r* = 0.72, *p* < 0.0001; Figure 3F,G). In addition, a positive correlation (*r* = 0.69, *p* < 0.0001) was observed between WBCs and D‐dimer in female patients (Figure 3G), but the correlation was much weaker in males (*r* = 0.49, *p* < 0.0001; Figure 3F).

When the immune system reacted with the SARS‐CoV‐2 virus during the second week of hospitalization, the WBC and CRP levels were elevated in a positively correlated manner. Elevation of blood leukocytes and CRP may be used as an indicator of the severity of COVID‐19.^25^ During the course of COVID‐19, elevation of leukocyte count and CRP is common among hospitalized COVID‐19 patients. Although elevated D‐dimer levels are consistently observed, their gradual increase during the course of the disease is particularly associated with disease worsening.^26^ The difference in correlation strength between D‐dimer and WBCs regarding male and female patients may be a second factor, in addition to age, to explain the previously mentioned high rate of death among females (see Figure 2).

**Figure 4:**
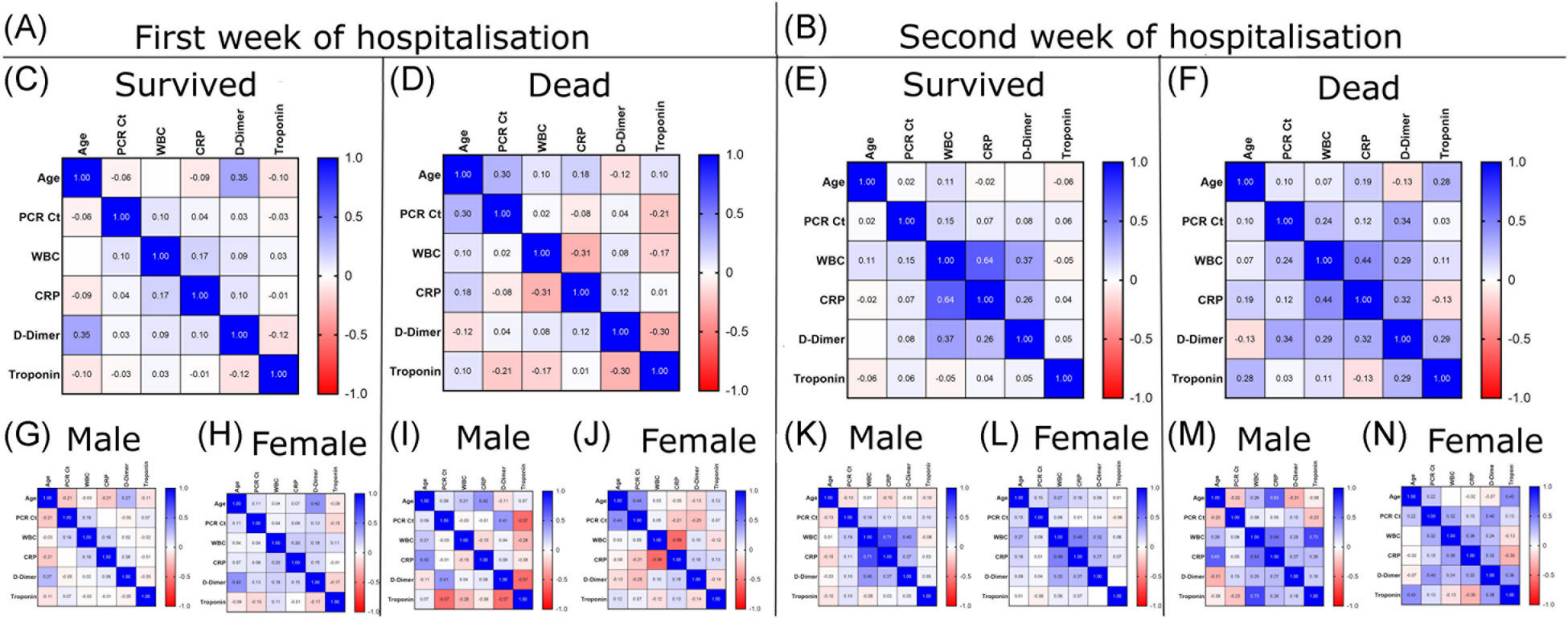
Correlation between studied parameters; Age, PCR‐Ct, WBC, CRP, d‐dimer and troponin at the first week of COVID‐19 patient admission (A) in survived (C); male (G), female (H) and dead COVID‐19 patients (D); male (I), female (J). Also at the second week of the patient admission (B) in survived (E); male (K), female (L) and dead COVID‐19 patients (F); male (M), female (N). CRP, C‐reactive protein; PCR‐Ct, polymerase chain reaction–cycle threshold; WBC, white blood cell

When the results were analyzed by category (survivors and deceased), neither survivors nor deceased patients showed any correlation among the studied parameters during their first week of hospital admission (Figure 4A). In the second week of the hospitalization, however, we found a positive correlation (*r* = 0.71, *p* < 0.0001) between WBCs and CRP in male survivors (Figure 3K). CRP is a universal inflammatory factor that has been evaluated under many conditions^27^ and recently, it has been found that WBC count, coupled with CRP, is involved in the prognosis of COVID‐19.^28^, ^29^

A strong positive correlation (*r* = 0.73, *p* = 0.007) was observed between WBCs and troponin in deceased male patients (Figure 4M), indicating a vital role for troponin in predicting death from COVID‐19. Troponin levels are a well‐established marker of myocardial injury,^30^ and early studies in patients with COVID‐19 reported that elevated plasma troponin levels were common and associated with a more severe clinical course and increased in‐hospital death.^31^, ^32^, ^33^, ^34^

**Figure 5:**
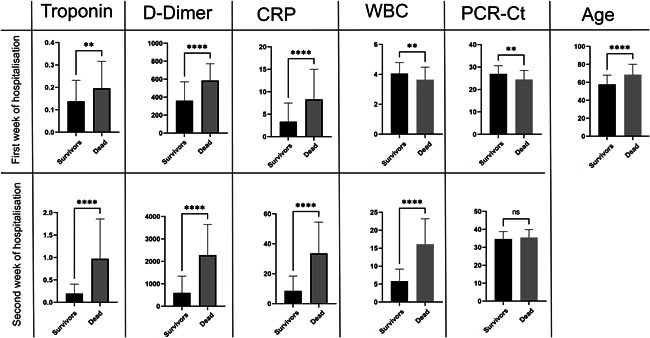
Differences in parameters between survivors and the dead COVID‐19 patients at first and second week of the COVID‐19 disease. CRP, C‐reactive protein; PCR‐Ct, polymerase chain reaction–cycle threshold; WBC, white blood cell. NS, 0.1234; **0.0021; ****<0.0001.

There was a highly significant difference in age (*p* < 0.0001, 10.76 ± 2.102 years) between survivors (57.8 years) and those who died (68.6 years; Figure 5), supporting the likelihood that age may play a role in the rate of death. Reports from different countries refer to a greater risk of death and high mortality rate among older patients with COVID‐19, in comparison with younger patients.^35^, ^36^, ^37^

In the first week of patient admission, there was a highly significant difference (*p* < 0.0001) between both groups in D‐dimer (226.2 ± 41.53 ng/ml) and CRP (5.000 ± 0.9403 mg/L) levels. d‐dimer elevation has been linked to coagulopathy and, with disseminated intravascular coagulation,^12^, ^38^ can lead to COVID‐19‐associated coagulopathy,^39^, ^40^ subsequently developing into capillary microthrombosis, as observed in post‐mortem studies.^41^ In addition, CRP can be considered an early indicator of pneumonia and the severity of COVID‐19.^42^, ^43^

In the first week of admission, there were also significant differences between both groups regarding troponin (*p* = 0.0038; 0.05817 ± 0.01984), WBCs (*p* = 0.0074; −0.4137 ± 0.1526), and PCR‐Ct (*p* = 0.001; −2.502 ± 0.7484; Figure 5). Levels of d‐dimer, CRP, troponin, and viral load were higher in patients who died than in survivors, whereas WBC count was higher among survivors. Troponin increases indicate myocardial injury and have been correlated positively with mortality,^44^ and some studies have considered it a predictor of death in COVID‐19 patients.^45^ Although a number of studies have shown that immune cells may play a serious role in COVID‐19 severity and susceptibility,^46^, ^47^, ^48^ the relation between WBC levels and severity of COVID‐19 remains unclear. Interestingly, for unknown reasons, the viral load of deceased patients in their first week of admission was significantly lower than that of survivors.^49^

In the second week of hospitalization, all studied parameters differed highly significantly (*p* < 0.0001) between survivors and deceased COVID‐19 patients, with the exception of PCR‐Ct. Mean differences between survivors and those who died were 0.7782 ± 0.08275 for troponin, 1685 ± 176.4 for d‐dimer, 25.10 ± 2.500 for CRP, and 10.32 ± 0.8450 for WBC count. When the levels of troponin, D‐dimer, CRP, and WBCs had returned to normal in the survivors, all remained elevated in the patients who died. If we take into account that the PCR‐Ct value was negative for both categories, it may be concluded that secondary infection^50^ and multiorgan dysfunction^51^ caused by the SARS‐CoV‐2 virus were possible causes of death. Elevation of cardiac injury and coagulation pathways may be relevant in defining the risk of mortality related to elevated troponin levels in patients with SARS‐CoV‐2.^52^

The current study showed that there were no significant differences in any parameters between male and female patients in both weeks of admission (Figure S1).

### 3.2 Second study

**Figure 6:**
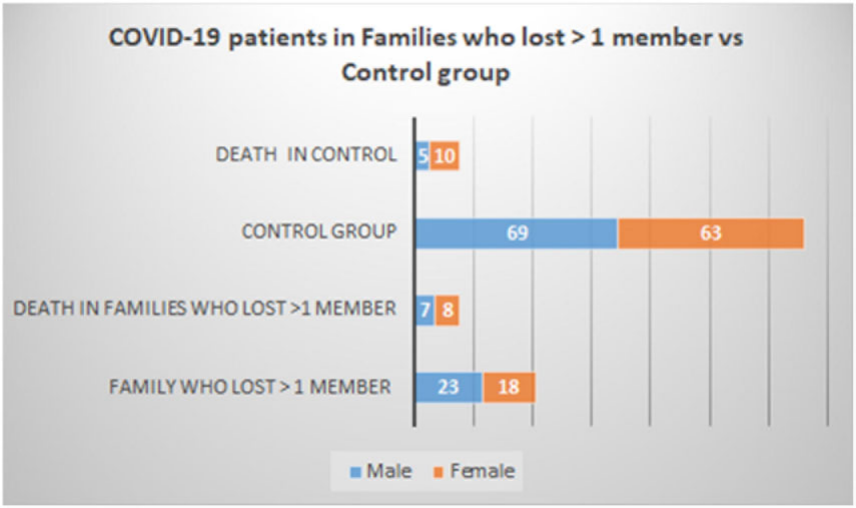
COVID‐19 patients were re‐categorized into two category: first, families who lost >1 member due to COVID‐19 (41 patients; male, 23 and female, 18). Death number in this group was 15 (male, 7 and female, 8). Second, the control (132 patients; male, 69 and female, 63), this group lost 5 male and 10 females)

In the second part of this study, COVID‐19 patients were re‐categorized into two groups. The first group comprised seven families (41 patients; 23 males, 18 females) that lost more than one member due to COVID‐19, with a total of 15 deaths recorded (seven males, eight females). The second group, which was the control (132 patients; 69 males, 63 females), also recorded 15 deaths (five males, 10 females; Figure 6).

**Figure 7:**
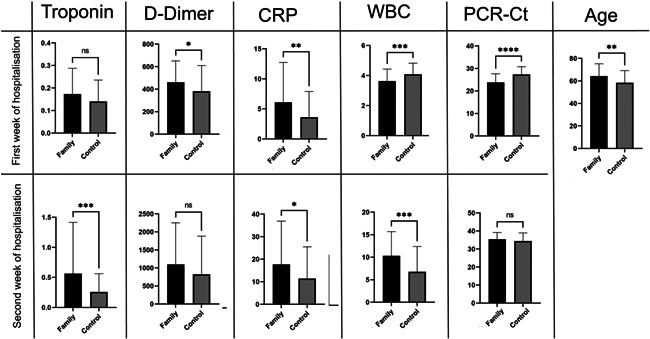
The differences between families with >1 death and control group in some parameter value in the first and the second week of COVID‐19 disease first and second week of the COVID‐19 disease. CRP, C‐reactive protein; PCR‐Ct, polymerase chain reaction–cycle threshold; WBC, white blood cell. NS, 0.1234; *0.0332; **0.0021; ***0.0002; ****<0.0001.

There was a significant difference in age between families in which more than one death occurred and the COVID‐19 patient control group (*p* = 0.003, −5.911 ± 1.946; Figure 7). Detailed data related to parameters correlated with families who recorded more than one death or the control group during the first and second week of patient hospitalization, taking into account gender differences, are presented in Figure S3.

In the first week of patient admission, both groups differed significantly in PCR‐Ct (*p* < 0.0001, 3.542 ± 0.6321), WBC count (*p* = 0.001, 0.4515 ± 0.1344), CRP (*p* = 0.006, −2.484 ± 0.8835), and d‐dimer (*p* = 0.044, −80.29 ± 39.58), but there were no significant differences in troponin levels (Figure 7).

In the second week of hospitalization, patients in families with more than one death differed highly significantly from the control group in relation to troponin (*p* = 0.0006, −0.3055 ± 0.08769), WBC count (*p* = 0.0005, −3.517 ± 0.9938), and CRP (*p* = 0.024, −6.273 ± 2.765). Conversely, there were no significant differences between the groups in d‐dimer and PCR‐Ct (Figure 7).

Levels of WBC, CRP, d‐dimer, and troponin (not clearly significant) during the first week of hospital admission differed between families that lost more than one member and the control group (Figure 7). These differences remained and appeared more clearly in the second week, especially the differences in troponin and D‐dimer. Family variation could explain why some families lost more members than others. The difference in death rate among families could be due specifically to some genetic variations among these families. For example, the predicted binding peptides for SARS‐CoV‐2 were found in patients with the HLA gene allele HLA‐B*46:01,^53^ suggesting that individuals in families harboring this allele may be particularly vulnerable to COVID‐19.^54^ Additionally, the HLA‐C*05 allele was correlated with COVID‐19 mortality in an ecological study.^55^

The host's genetic background influencing the susceptibility and outcome of multiple infectious diseases has been previously reported^56^, ^57^ and a global project has been started to understand the pathogenesis of COVID‐19, especially severe cases in young people.^57^ In addition, the “COVID‐19 Host Genetics Initiative” has been launched to bring the human genetics community together to identify the genetic determinants of COVID‐19 susceptibility, severity, and outcomes.^58^ Here, family genetic sequencing studies are needed to investigate increased troponin and WBC levels in families who are highly susceptible to death.

To conclude, the current study observed that levels of troponin, D‐dimer, CRP, and WBCs were significantly higher in COVID‐19 patients who died than in COVID‐19 survivors. High troponin and WBC levels, especially within the second week of admission, were considerably associated with families that lost more than one member, when compared with the unrelated COVID‐19 patient control.

## CONFLICT OF INTERESTS

The authors declare that there are no conflict of interests.

## ETHICS STATEMENT

The protocol was approved by the Ethics Licensing Committee of the Kalar Technical College at the Sulaimani Polytechnic University Committee (No. 05 on January 14, 2021) and followed the Declaration of Helsinki.

## AUTHOR CONTRIBUTIONS

Ayad M. Ali performed lab work and data collection; Hassan M. Rostam analyzed the data and wrote the results; Kameran M. Ali, Chalak M. Noori, Mohammed H. Fatah, and Hassan M. Tawfeeq contributed in the writing of the manuscript. All authors approved the final version of the manuscript.

## Supporting information

Supporting information.

## Data Availability

The data that support the findings of this study are available from the corresponding author upon reasonable request.

## References

[1] Zhu N , Zhang D , Wang W , et al. A novel coronavirus from patients with pneumonia in China, 2019. N Engl J Med. 2020;382(8):727‐733. 31978945 10.1056/NEJMoa2001017 PMC7092803

[2] Wu F , Zhao S , Yu B , et al. A new coronavirus associated with human respiratory disease in China. Nature. 2020;579(7798):265‐269. 32015508 10.1038/s41586-020-2008-3 PMC7094943

[3] WHO . WHO Coronavirus (COVID‐19) Dashboard; 2021. Accessed June 12, 2021. https://covid19.who.int/?gclid=Cj0KCQjwweyFBhDvARIsAA67M703RW_Ot4bPRwGTcP_GSFoRJwZl4u2UIHZK0SgQf_wR7KPuVjMwi5MaApSJEALw_wcB

[4] Sharma R , Agarwal M , Gupta M , Somendra S , Saxena S.K. Clinical characteristics and differential clinical diagnosis of novel coronavirus disease 2019 (COVID‐19), in coronavirus disease 2019 (COVID‐19). In: Saxena SK , ed. Epidemiology, Pathogenesis, Diagnosis, and Therapeutics. Springer Singapore; 2020:55‐70. https://link.springer.com/content/pdf/10.1007%252F978-981-15-4814-7.pdf#page=69

[5] Wu D , Wu T , Liu Q , Yang Z . The SARS‐CoV‐2 outbreak: what we know. Int J Infect Dis. 2020;94:44‐48. 32171952 10.1016/j.ijid.2020.03.004 PMC7102543

[6] Mokhtari T , Hassani F , Ghaffari N , Ebrahimi B , Yarahmadi A , Hassanzadeh G . COVID‐19 and multiorgan failure: a narrative review on potential mechanisms. J Mol Histol. 2020;51(6):613‐628. 33011887 10.1007/s10735-020-09915-3 PMC7533045

[7] Manson JJ , Crooks C , Naja M , et al. COVID‐19‐associated hyperinflammation and escalation of patient care: a retrospective longitudinal cohort study. Lancet Rheumatol. 2020;2(10):e594‐e602. 32864628 10.1016/S2665-9913(20)30275-7 PMC7442426

[8] Mousavi SA , Rad S , Rostami T , et al. Hematologic predictors of mortality in hospitalized patients with COVID‐19: a comparative study. Hematology. 2020;25(1):383‐388. 33124971 10.1080/16078454.2020.1833435

[9] Ponti G , Maccaferri M , Ruini C , Tomasi A , Ozben T . Biomarkers associated with COVID‐19 disease progression. Crit Rev Clin Lab Sci. 2020;57(6):389‐399. 32503382 10.1080/10408363.2020.1770685 PMC7284147

[10] Yu M , Cheng X . Cardiac injury is independently associated with mortality irrespective of comorbidity in hospitalized patients with coronavirus disease 2019. Cardiol J. 2020;27(5):472‐473. 33165894 10.5603/CJ.2020.0150 PMC8078968

[11] Nishiga M , Wang DW , Han Y , Lewis DB , Wu JC . COVID‐19 and cardiovascular disease: from basic mechanisms to clinical perspectives. Nat Rev Cardiol. 2020;17(9):543‐558. 32690910 10.1038/s41569-020-0413-9 PMC7370876

[12] Tang N , Li D , Wang X , Sun Z . Abnormal coagulation parameters are associated with poor prognosis in patients with novel coronavirus pneumonia. J Thromb Haemostasis. 2020;18(4):844‐847. 32073213 10.1111/jth.14768 PMC7166509

[13] Hu D , Lou X , Meng N , et al. Influence of age and gender on the epidemic of COVID‐19. Wien Klin Wochenschr. 2021;133(7):321‐330. 33547492 10.1007/s00508-021-01816-z PMC7864622

[14] Lorente L , Martín MM , Franco A , et al. HLA genetic polymorphisms and prognosis of patients with COVID‐19. Medicina Intensiva. 2021;45(2):96‐103. 38620408 10.1016/j.medin.2020.08.004 PMC7474921

[15] National Health Commission , National Administration of Traditional Chinese Medicine . Translation: diagnosis and treatment protocol for novel coronavirus pneumonia (trial version 7). Infect Microbes Dis. 2020;2:2. 10.1097/IM9.0000000000000022 PMC7227203 40479241

[16] Ali KM , Ali AM , Tawfeeq HM , Figueredo GP , Rostam HM . Hypoalbuminemia in patients following their recovery from severe coronavirus disease 2019. J Med Virol. 2021. 93(7):4532‐4536. 33830538 10.1002/jmv.27002 PMC8250600

[17] Ali AM , Ali KM , Fatah MH , Tawfeeq HM , Rostam HM . SARS‐CoV‐2 reinfection in patients negative for immunoglobulin G following recovery from COVID‐19. New Microbes and New Infections. 2021;43:100926. 10.1016/j.nmni.2021.100926 34367645 PMC8327640

[18] GOV.UK . Guidance: cycle threshold (Ct) in SARS‐CoV‐2 RT‐PCR; 2021. Accessed August 19, 2021. https://www.gov.uk/government/publications/cycle-threshold-ct-in-sars-cov-2-rt-pcr.

[19] Jin J‐M , Bai P , He W , et al. Gender differences in patients with COVID‐19: focus on severity and mortality. Front Public Health. 2020;8:152. 32411652 10.3389/fpubh.2020.00152 PMC7201103

[20] Li Q , Guan X , Wu P , et al. Early transmission dynamics in Wuhan, China of novel coronavirus–infected pneumonia. N Engl J Med. 2020;382(13):1199‐1207. 31995857 10.1056/NEJMoa2001316 PMC7121484

[21] Zhang J‐j , Dong X , Cao YY , et al. Clinical characteristics of 140 patients infected with SARS‐CoV‐2 in Wuhan, China. Allergy. 2020;75(7):1730‐1741. 32077115 10.1111/all.14238

[22] Chen N , Zhou M , Dong X , et al. Epidemiological and clinical characteristics of 99 cases of 2019 novel coronavirus pneumonia in Wuhan, China: a descriptive study. Lancet. 2020;395(10223):507‐513. 32007143 10.1016/S0140-6736(20)30211-7 PMC7135076

[23] Huang C , Wang Y , Li X , et al. Clinical features of patients infected with 2019 novel coronavirus in Wuhan, China. The Lancet. 2020;395(10223):497‐506. 10.1016/s0140-6736(20)30183-5 PMC7159299 31986264

[24] Chaussabel D , Pascual V , Banchereau J . Assessing the human immune system through blood transcriptomics. BMC Biol. 2010;8(1):84. 20619006 10.1186/1741-7007-8-84 PMC2895587

[25] Yamada T , Wakabayashi M , Yamaji T , et al. Value of leukocytosis and elevated C‐reactive protein in predicting severe coronavirus 2019 (COVID‐19): a systematic review and meta‐analysis. Clin Chim Acta. 2020;509:235‐243. 32533986 10.1016/j.cca.2020.06.008 PMC7832771

[26] Terpos E , Ntanasis‐Stathopoulos I , Elalamy I , et al. Hematological findings and complications of COVID‐19. Am J Hematol. 2020;95(7):834‐847. 32282949 10.1002/ajh.25829 PMC7262337

[27] Vermeire S , Van Assche G , Rutgeerts P . C‐reactive protein as a marker for inflammatory bowel disease. Inflamm Bowel Dis. 2004;10(5):661‐665. 15472532 10.1097/00054725-200409000-00026

[28] Liu F , Li L , Xu M , et al. Prognostic value of interleukin‐6, C‐reactive protein, and procalcitonin in patients with COVID‐19. J Clin Virol. 2020;127:104370. 32344321 10.1016/j.jcv.2020.104370 PMC7194648

[29] Wang L . C‐reactive protein levels in the early stage of COVID‐19. Med Mal Infect. 2020;50(4):332‐334. 32243911 10.1016/j.medmal.2020.03.007 PMC7146693

[30] McCarthy CP , Raber I , Chapman AR , et al. Myocardial injury in the era of high‐sensitivity cardiac troponin assays: a practical approach for clinicians. JAMA Cardiol. 2019;4(10):1034‐1042. 31389986 10.1001/jamacardio.2019.2724

[31] Shi S , Qin M , Shen B , et al. Association of cardiac injury with mortality in hospitalized patients with COVID‐19 in Wuhan, China. JAMA Cardiology. 2020;5(7):802‐810. 32211816 10.1001/jamacardio.2020.0950 PMC7097841

[32] Wei JF , Huang FY , Xiong TY , et al. Acute myocardial injury is common in patients with COVID‐19 and impairs their prognosis. Heart. 2020;106(15):1154‐1159. 32354798 10.1136/heartjnl-2020-317007 PMC7398466

[33] Guo T , Fan Y , Chen M , et al. Cardiovascular implications of fatal outcomes of patients with coronavirus disease 2019 (COVID‐19). JAMA Cardiol. 2020;5(7):811‐818. 32219356 10.1001/jamacardio.2020.1017 PMC7101506

[34] Zhou F , Yu T , Du R , et al. Clinical course and risk factors for mortality of adult inpatients with COVID‐19 in Wuhan, China: a retrospective cohort study. Lancet. 2020;395(10229):1054‐1062. 32171076 10.1016/S0140-6736(20)30566-3 PMC7270627

[35] Onder G , Rezza G , Brusaferro S . Case‐Fatality rate and characteristics of patients dying in relation to COVID‐19 in Italy. JAMA. 2020;323(18):1775‐1776. 32203977 10.1001/jama.2020.4683

[36] Richardson S , Hirsch JS , Narasimhan M , et al. Presenting characteristics, comorbidities, and outcomes among 5700 patients hospitalized with COVID‐19 in the New York City Area. JAMA. 2020;323(20):2052‐2059. 32320003 10.1001/jama.2020.6775 PMC7177629

[37] Inciardi RM , Adamo M , Lupi L , et al. Characteristics and outcomes of patients hospitalized for COVID‐19 and cardiac disease in Northern Italy. Eur Heart J. 2020;41(19):1821‐1829. 32383763 10.1093/eurheartj/ehaa388 PMC7239204

[38] Tang N , Bai H , Chen X , Gong J , Li D , Sun Z . Anticoagulant treatment is associated with decreased mortality in severe coronavirus disease 2019 patients with coagulopathy. J Thromb Haemostasis. 2020;18(5):1094‐1099. 32220112 10.1111/jth.14817 PMC9906401

[39] Helms J , Severac F , Merdji H , Anglés‐Cano E , Meziani F . Prothrombotic phenotype in COVID‐19 severe patients. Intensive Care Med. 2020;46(7):1502‐1503. 32435823 10.1007/s00134-020-06082-7 PMC7237619

[40] Klok FA , Kruip M , van der Meer N , et al. Incidence of thrombotic complications in critically ill ICU patients with COVID‐19. Thromb Res. 2020;191:145‐147. 32291094 10.1016/j.thromres.2020.04.013 PMC7146714

[41] Xie X , Zhong Z , Zhao W , Zheng C , Wang F , Liu J . Chest CT for typical coronavirus disease 2019 (COVID‐19) pneumonia: relationship to negative RT‐PCR testing. Radiology, 2020. 296(2):E41‐E45. 32049601 10.1148/radiol.2020200343 PMC7233363

[42] Pimentel MAF , Redfern OC , Hatch R , Young JD , Tarassenko L , Watkinson PJ . Trajectories of vital signs in patients with COVID‐19. Resuscitation. 2020;156:99‐106. 32918984 10.1016/j.resuscitation.2020.09.002 PMC7481128

[43] Cekerevac I , Turnic TN , Draginic N , et al. Predicting severity and intrahospital mortality in COVID‐19: the place and role of oxidative stress. Oxid Med Cell Longevity. 2021;2021:6615787. 10.1155/2021/6615787 PMC8019372 33854695

[44] Zou F , Qian Z , Wang Y , Zhao Y , Bai J . Cardiac injury and COVID‐19: a systematic review and meta‐analysis. CJC Open. 2020;2(5):386‐394. 32838255 10.1016/j.cjco.2020.06.010 PMC7308771

[45] Majure DT , Gruberg L , Saba SG , et al. Usefulness of elevated troponin to predict death in patients with COVID‐19 and myocardial injury. Am J Cardiol. 2021;138:100‐106. 33058800 10.1016/j.amjcard.2020.09.060 PMC7550867

[46] Qin C , Zhou L , Hu Z , et al. Dysregulation of immune response in patients with coronavirus 2019 (COVID‐19) in Wuhan, China. Clin Infect Dis. 2020;71(15):762‐768. 32161940 10.1093/cid/ciaa248 PMC7108125

[47] Chen R , Sang L , Jiang M , et al. Longitudinal hematologic and immunologic variations associated with the progression of COVID‐19 patients in China. J Allergy Clin Immunol. 2020;146(1):89‐100. 32407836 10.1016/j.jaci.2020.05.003 PMC7212968

[48] Vitte J , Diallo AB , Boumaza A , et al. A granulocytic signature identifies COVID‐19 and its severity. J Infect Dis. 2020;222(12):1985‐1996. 32941618 10.1093/infdis/jiaa591 PMC7543529

[49] Walsh KA , Jordan K , Clyne B , et al. SARS‐CoV‐2 detection, viral load and infectivity over the course of an infection. J Infect. 2020;81(3):357‐371. 32615199 10.1016/j.jinf.2020.06.067 PMC7323671

[50] Manohar P , Loh B , Athira S , et al. Secondary bacterial infections during pulmonary viral disease: phage therapeutics as alternatives to antibiotics? Front Microbiol. 2020;11:1434. 32733404 10.3389/fmicb.2020.01434 PMC7358648

[51] Ayoubkhani D , Khunti K , Nafilyan V , et al. Post‐covid syndrome in individuals admitted to hospital with covid‐19: retrospective cohort study. BMJ. 2021;372:n693. 33789877 10.1136/bmj.n693 PMC8010267

[52] Mengozzi A , Georgiopoulos G , Falcone M , et al. The relationship between cardiac injury, inflammation and coagulation in predicting COVID‐19 outcome. Sci Rep. 2021;11(1):6515. 33753759 10.1038/s41598-021-85646-z PMC7985490

[53] Nguyen A , David JK , Maden SK , et al. Human leukocyte antigen susceptibility map for severe acute respiratory syndrome coronavirus 2. J Virol. 2020;94(13):e00510‐e00520. 32303592 10.1128/JVI.00510-20 PMC7307149

[54] Lin M , Tseng HK , Trejaut JA , et al. Association of HLA class I with severe acute respiratory syndrome coronavirus infection. BMC Med Genet. 2003;4(1):9. 12969506 10.1186/1471-2350-4-9 PMC212558

[55] Sakuraba A , Haider H , Sato T . Population difference in allele frequency of HLA‐C*05 and its correlation with COVID‐19 mortality. Viruses. 2020;12(11):1333. 10.3390/v12111333 PMC7699862 33233780

[56] Fricke‐Galindo I , Falfán‐Valencia R . Genetics insight for COVID‐19 susceptibility and severity: a review. Front Immunol. 2021;12:1057. 10.3389/fimmu.2021.622176 PMC8047200 33868239

[57] Casanova JL , Su HC . A global effort to define the human genetics of protective immunity to SARS‐CoV‐2 infection. Cell. 2020;181(6):1194‐1199. 32405102 10.1016/j.cell.2020.05.016 PMC7218368

[58] COVID‐19 Host Genetics Initiative . The COVID‐19 Host Genetics Initiative, a global initiative to elucidate the role of host genetic factors in susceptibility and severity of the SARS‐CoV‐2 virus pandemic. Eur J Human Genet. 2020;28(6):715‐718. 32404885 10.1038/s41431-020-0636-6 PMC7220587

