# Supplementary material for: Serum troponin, D‐dimer, and CRP level in severe coronavirus (COVID‐19) patients
Source: Immun Inflamm Dis. 2021 Dec 22;10(3):e582. doi: 10.1002/iid3.582 (PMC8926504; doi:10.1002/iid3.582)
Supplement: Supplementary file 1 — Supporting information. [file IID3-10-e582-s001.docx]

**Supplementary data**

**Cardiac Troponin I and D-Dimer Level in Severe Coronavirus (COVID-19) Patient**

| First week of hospitalisation | | | | | Age  Male/female |
| --- | --- | --- | --- | --- | --- |
| Troponin  male /female | D-dimer  Male/female | CRP  Male/female | WBC  Male/female | PCR-Ct  Male/female |  |
| 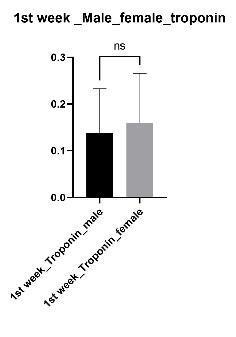 | 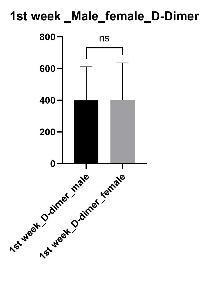 | 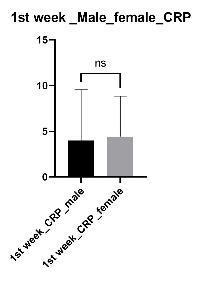 | 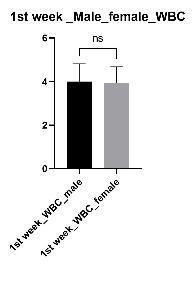 | 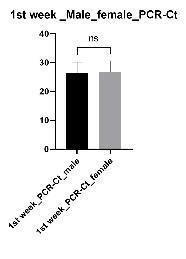 | 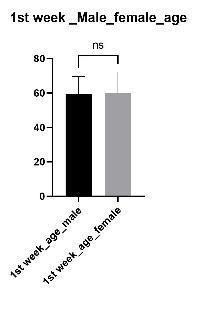 |
| Second week of hospitalisation | | | | |  |
| Troponin  male /female | D-dimer  Male/female | CRP  Male/female | WBC  Male/female | PCR-Ct  Male/female |  |
| 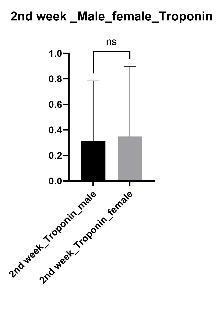 | 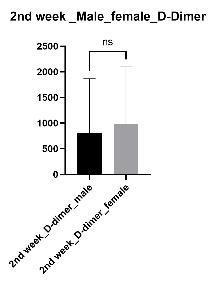 | 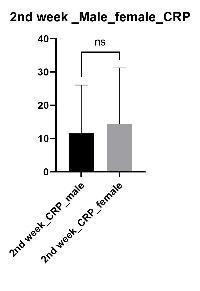 | 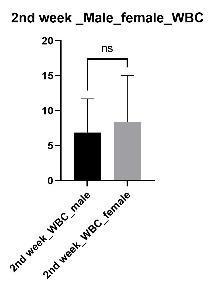 | 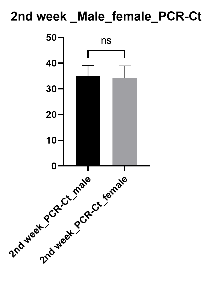 |  |

**Supplementary Figure 1** Differences between male and female in parameters at the first and second week of disease in whole COVID-19 studded patients

In the first week of the patient’s admission, studied parameters were not correlated to each other in both COVID-19 patient study groups; families with > 1 dead and COVID-19 patient control group (Supplementary Figure 2A). In the second week of the disease it has been noticed that troponin in the family> 1 dead group correlated (r=0.7) positively with D-dimer (Supplementary Figure 2E). The correlation was higher in females(r=0.75)( Supplementary Figure 2K) than male (r=0.65) (Supplementary Figure 2L)in the same group. D-dimer in males in the family with >1 dead had a strong correlation with CRP (r=79) and WBC (r=0.77)( Supplementary Figure 2K).

During the second week of the patients admission, WBC in COVID-19 control group was correlated positively with CRP in both genders (male, r=0.81(Supplementary Figure 2M) and female, 0.76 (Supplementary Figure 2N)). In addition, WBC in females in the same group experienced a strong positive correlation (r=0.83) with D-dimer (Supplementary Figure 2N).

| **A** First week of hospitalisation | | | | **B**  Second week of hospitalisation | | | |
| --- | --- | --- | --- | --- | --- | --- | --- |
| **C** Family with > 1 dead | | **D** Control | | **E** Family with > 1 dead | | **F**  Control | |
| 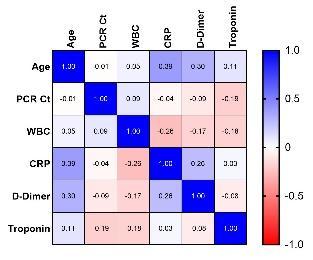 | | 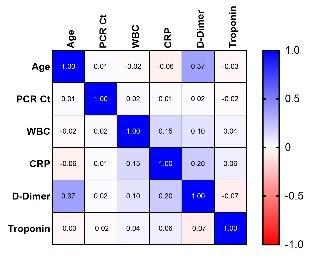 | | 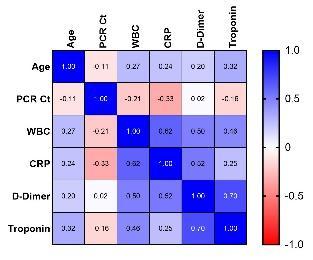 | | 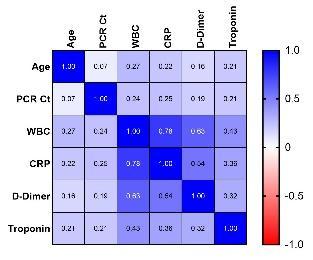 | |
| **G** Male | **H**  Female | **I** Male | **J** Female | **K**  Male | **L** Female | **M** Male | **N**  Female |
| 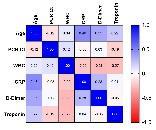 | 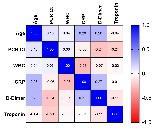 | 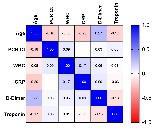 | 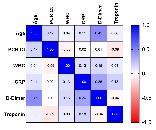 | 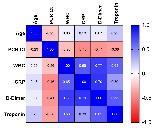 | 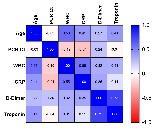 | 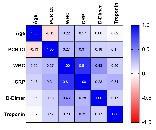 | 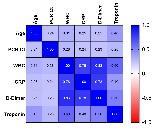 |

**Supplementary Figure 2:** Correlation between studied parameters; Age, PCR-Ct, WBC, CRP, D-dimer and troponin at the 1^st^ week (A) in family group with> 1 COVID-19 death (C); male (G), female (H) and COVID-19 control patients (D); male (I), female (J). Also at the 2^nd^ week (B) in family > 1 COVID-19 death (E); male (K), female (L) and COVID-19 control patients (F); male (M), female (N).

During the first week of the patient admission to the hospital, survivor patients from families who lost > 1 member and the control group have not experienced any correlation. On the other hand, troponin of dead patients (male (Supplementary Figure 3K) and females (Supplementary Figure 3L)) in the families who lost >1 patient had a negative correlation(r=-0.75) D-dimer (Supplementary Figure 3E). Troponin in dead male in the same group correlated negatively (-0.74) with PCR-Ct (Supplementary Figure 3K).However, in dead female patients in the same group we found a high negative correlation (r=-0.84) between CRP and WBC.

During the first week of the admission, troponin in male dead patients in COVID-19 control group had a strong negative correlation (r=-0.80) with D-dimer. Also, male in the same group experienced a negative correlation (r=-0.70) between WBC and PCR-Ct (Supplementary Figure 3M). However, WBC in dead females COVID-19 control group had a positive correlation with PCR-Ct. D-dimer had a positive correlation (r=0.79) with CRP in females at the same group (Supplementary Figure 3N).

Correlation between studied parameters the second week of admission in COVID-19 survivors (A2) in family with> 1 COVID-19 death (Supplementary Figure 3C2); control group(Supplementary Figure 3G2), female (Supplementary Figure 3H2) and COVID-19 control patients (Supplementary Figure 3D2); male (Supplementary Figure 3I2), female (Supplementary Figure 3J2). Also, correlation between parameters in the 2^nd^ week in dead COVID-19 patients (B2) in family > 1 COVID-19 death (Supplementary Figure 3E2); male (Supplementary Figure 3 K2), female (Supplementary Figure 3L2) and COVID-19 control patients (Supplementary Figure 3F2); male (Supplementary Figure 3M2), female (Supplementary Figure 3N2).

In the second week of the COVID-19 patient admission to the hospital, the male COVID-19 survivors from families who lost more than one member had a high correlation between; D-dimer and CRP, (r=0.94); D-dimer and WBC, (r=0.85); CRP and WBC (r=0.75)( Supplementary Figure 3G2). However, there was no correlation between studied parameters in female patients in the same group(Supplementary Figure 3H2).

In the COVID-19 survivor control patients, the best correlation (positive correlation) (r=0.68) was found between CRP and WBC (Supplementary Figure 3D2), the correlation was higher in male (r=0.71, Supplementary Figure 3I2) than female (r=0.66, Supplementary Figure 3J2) at the same group.

Figure 8K2 shows correlation with power r=0.67 between Troponin and WBC during the second week of the admission in dead male in the families with more than 1 dead, while the female in the same group had a correlation (r=0.68) between troponin and D-dimer (Supplementary Figure 3L2).

During the 2^nd^ week of admission in COVID-19 patient controls, troponin in male patients had a strong positive correlation with WBC (r=0.97) and CRP(r=0.97), Also CRP a high positive correlation (r=0.95) with WBC (Supplementary Figure 3M2). However, females in the same group had only a positive correlation (r=0.76) between age and troponin parameter (Supplementary Figure 3N2).

| **A** First week of hospitalisation in survivors | | | | **B**  First week of hospitalisation in dead | | | |
| --- | --- | --- | --- | --- | --- | --- | --- |
| **C** family with > 1 dead | | **D** control group | | **E** family with > 1 dead | | **F**  control group | |
| 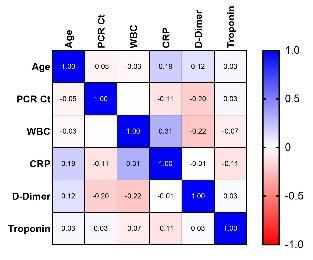 | | 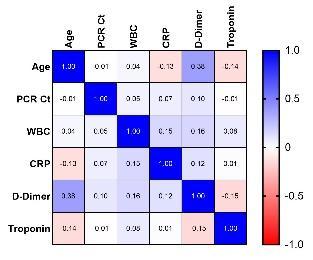 | | 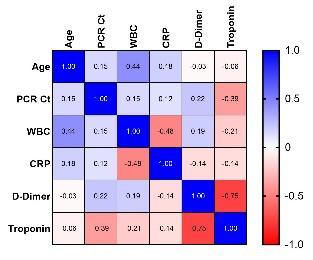 | | 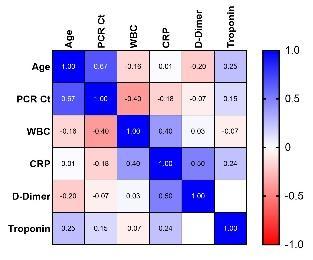 | |
| **G** Male | **H**  Female | **I** Male | **J** Female | **K**  Male | **L** Female | **M** Male | **N**  Female |
| 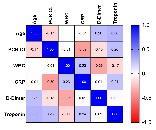 | 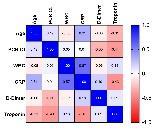 | 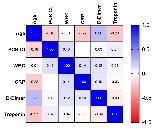 | 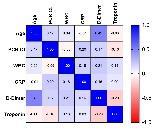 | 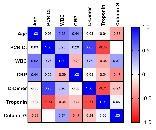 | 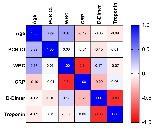 | 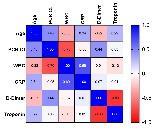 | 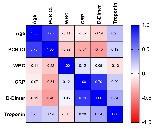 |
| **A2** Second week of hospitalisation in survivors | | | | **B2**  Second week of hospitalisation in dead | | | |
| **C2** family with > 1 dead | | **D2** control group | | **E2** family with > 1 dead | | **F2**  control group | |
| 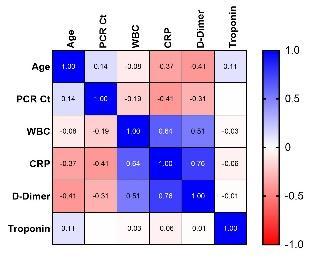 | | 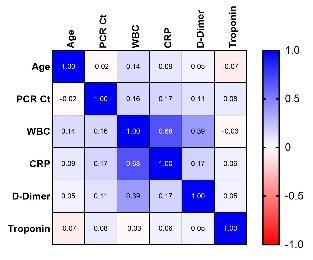 | | 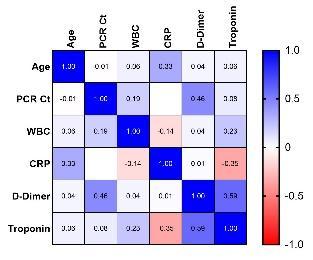 | | 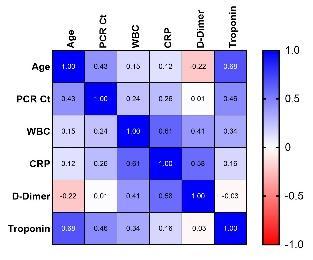 | |
| **G2** Male | **H2**  Female | **I2** Male | **J2** Female | **K2**  Male | **L2** Female | **M2** Male | **N2**  Female |
| 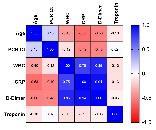 | 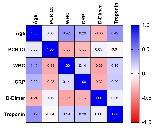 | 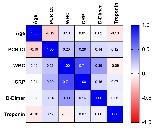 | 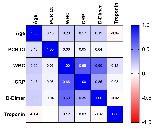 | 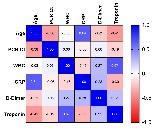 | 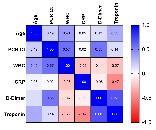 | 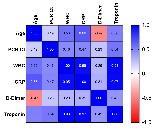 | 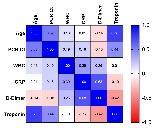 |

**Supplementary Figure 3:** Correlation between studied parameters; Age, PCR-Ct, WBC, CRP, D-dimer and troponin in the first week in COVID-19 survivors(A) in family with> 1 COVID-19 death (C); control group(G), female (H) and COVID-19 control patients (D); male (I), female (J). Also, correlation between parameters in the first week in dead COVID-19 patients (B) in family > 1 COVID-19 death (E); male (K), female (L) and COVID-19 control patients (F); male (M), female (N).
